# Posterior Semicircular Canal Benign Paroxysmal Positional Vertigo Presenting with Torsional Downbeating Nystagmus: An Apogeotropic Variant

**DOI:** 10.1155/2012/413603

**Published:** 2012-08-28

**Authors:** Paolo Vannucchi, Rudi Pecci, Beatrice Giannoni

**Affiliations:** Service of Audiology, Department of Oto-Neuro-Ophthalmology, Surgical Sciences, University of Florence, Viale Morgagni 85, 50100 Florence, Italy

## Abstract

The aim of this study is to verify the hypothesis that free-floating particles could sometimes localize into the distal portion of the non ampullary arm of the posterior semicircular canal (PSC) so that assuming the Dix-Hallpike's positions, the clot could move towards the ampulla eliciting a inhibitory torsional-down beating paroxysmal positional nystagmus (PPNy), instead of typical excitatory torsional-up beating PPNy. Among 45 patients with vestibular signs suggesting anterior semicircular canal paroxysmal positional vertigo (PPV), collected from February 2003 to August 2006, we detected a group of 6 subjects whose clinical findings showed a singular behaviour during follow-up. At the first check-up, all patients were submitted to different types of physical manoeuvres for ASC canalolithiasis. Patients were controlled during the same session and after one week. When we found that nystagmus was qualitatively changed we adopted the appropriate physical therapies for that sign. At a next check-up, after having performed some physical therapies, all patients had a typical PSC PPNy of the opposite side, with respect to that of the ASC initially diagnosed. Basing on these observations we conclude that PSC PPV, similarly to lateral semicircular canal PPV, could manifests in a apogeotropic variant.

## 1. Introduction

Paroxysmal positional vertigo (PPV) is the most frequently encountered peripheral vestibular syndrome. The underlying pathogenetic mechanism is, in most cases, canalolithiasis [1–3]. Canalolithiasis involves most frequently the posterior semicircular canal (PC); lateral semicircular canal (LC) and, overall, the anterior semicircular canal (AC) are interested more rarely [4–7].

Paroxysmal positional nystagmus (PPNy) has, in most cases, some paradigmatic features that leave no doubt about its clinical interpretation. However, in a restricted number of cases, PPNy appears with nonparadigmatic aspects that can mime a central vestibular pathology: such cases need neuroradiological investigations to clarify the diagnosis.

Nevertheless, with the deepening of knowledge about pathophysiological mechanisms of PPV, some nystagmic patterns, initially considered atypical, have been subsequently explained with the understanding of some mechanical drives. That is the case of LC PPV presenting with apogeotropic nystagmus. This form can be driven by a canalolithiasis (the otoconial mass being located initially into the ampullary end of the canal) or by a cupulothiasis (having the clot adhering to the cupula) [6, 8–10]. 

Moreover, apogeotropic LC PPV, presenting with apogeotropic paroxysmal positional nystagmus on both lateral side positions, can be converted into geotropic LC PPV, presenting with geotropic paroxysmal positional nystagmus on both lateral side positions, and viceversa sometimes only by repeating lateral side positionings [6].

**Figure 1:**
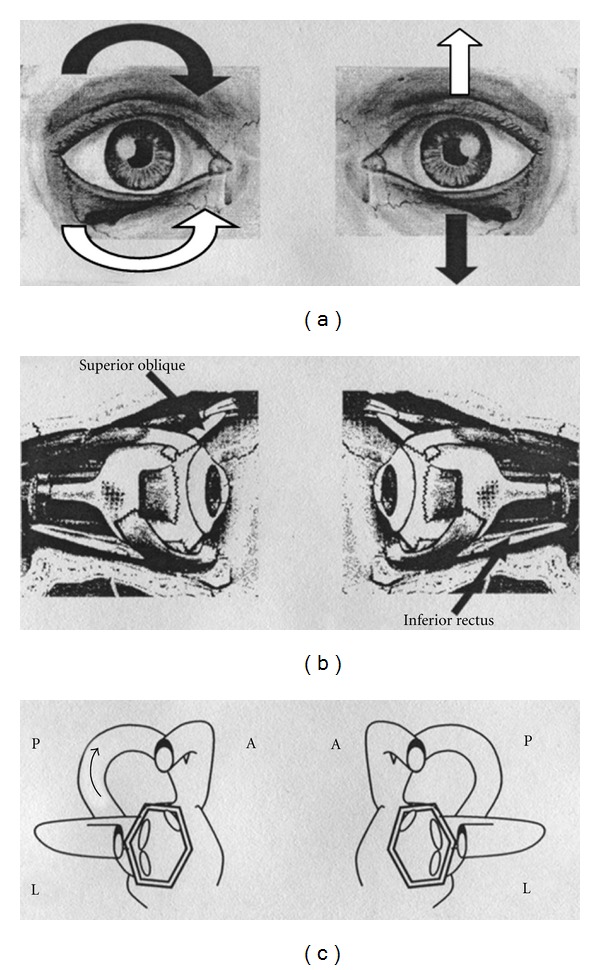
Paroxysmal positional nystagmus due to unilateral right posterior semicircular canal (PC) lithiasis (excitatory stimulus). (a): Black arrows indicate the direction of nystagmus slow phase in the two eyes; white arrows indicate the direction of nystagmus fast phase in the two eyes. (b): Arrows indicate the ocular muscles involved in nystagmus generation. (c): The two labyrinths; arrow indicates the endolymphatic flow within the interested canal. On the left side of the figure: the right eye and the right labyrinth; on the right side of the figure: the left eye and the left labyrinth. A: anterior semicircular canal; L: lateral semicircular canal; P: posterior semicircular canal.

Typical nystagmus due to PC excitation is vertical-torsional having the linear component of its fast phase directed upward (to the forehead) and the torsional one directed with the upper pole of the eye to the lower ear (being clockwise for the left PC and counterclockwise for the right PC, Figure 1). Typical PC PPNy is, therefore, geotropic, that is directed towards the earth in the provoking positions. The latter positions are the right and left Dix-Hallpike's positioning tests, respectively, for right and left PC PPV. 

Typical PC PPNy has a brief latency, it is paroxysmal, and it has a duration that usually does not overcome one minute; its direction reverses when the patient comes up to the sitting position, being therefore geotropic again. Pathogenetic mechanism underlying typical PC PPV is mostly that of canalolithiasis [1, 2].

In 1995 Agus et al. [11] first described an atypical PC PPNy, that is mainly vertical-torsional, but “reversed” in all its directional components when observed into provoking positions. The author speculated that, assuming the Dix-Hallpike's positions, such an ocular movement could be elicited by an ampullopetal endolymphatic flow produced by free-floating particles in the distal portion of the nonampullary branch of the PC.

Few years later Giannoni [12] theorized the existence of a PC PPV presenting with an apogeotropic nystagmus, even though she did not report patients' cases. 

AC PPV is characterized by a vertical-torsional positional nystagmus, with a predominant linear component directed, with the fast phase, downward; the torsional component is not always well evident and it is directed with the upper pole of the eye to the left ear for the left AC and to the right ear for the right AC. So the vertical component beats in the opposite direction to that of the PC PPV, and the torsonial component beats in the same direction of that expected for the involvement of the homolateral PC (being clockwise for the left AC and counterclockwise for the right AC). Provoking positions are the Dix-Hallpike's even if unilateral AC positional nystagmus is often elicited both in the right and left Dix-Hallpike's positions and into the central head hanging position. Pathogenetic mechanism underlying AC is thought to be, once more, that of a canalolithiasis. 

Recently, we collected some patients complaining of positional vertigo provoked by vertical positioning, with a vertical-torsional downbeating paroxysmal nystagmus thus suggesting AC PPV. At a next checkup these patients had a typical PC PPNy of the opposite side, with respect to that of the AC initially diagnosed. We hypothesized that free-floating particles could sometimes localize into the distal portion of the no ampullary arm of the posterior semicircular canal (PC) with a nystagmus similar to that of controlateral AC.

## 2. Materials and Methods

At the Unit of Audiology of the University of Florence, during the period that goes from February 2003 to August 2006, we collected 45 patients with vestibular symptoms and signs consistent with AC PPV. In the same period PPV was diagnosed in other 1374 subjects admitted to our clinic complaining of vertigo (918 PC PPV, 273 LC PPV, 183 PPV due to involvement of both canals).

Among patients with vestibular signs suggesting AC PPV we detected a group of 6 subjects whose clinical findings showed a singular behaviour during followup. 

Three out of these 6 patients had positional vertigo for the first time, while the other 3 had a prior documented PPV (in all cases a PC PPV).

Patients were all females, with a mean age of 50.33 years (minimum 41, maximum 64). A detailed otoneurologic history was collected in every case as well as an accurate remote and near general pathological and pharmacological history.

All patients underwent a microscope otologic inspection and an audiometric and impedance testing. Spontaneous-positional nystagmus was checked, with and without fixation (using Frenzel lenses and/or infrared videooculoscopy) into five positions (seated, supine, left and right sides, and head hanging) and into the two Dix-Hallpike's positioning tests. Enhanced head hanging position [13], mostly indicated to check AC PPV, was also tested in all cases. Gaze evoked and rebound nystagmus were checked both with and without fixation. Head shaking test (HST) and head thrust test (HTT) were performed in only one case: this because, having a suspicion of a PPV, we prefer not to carry out rapid head movements in order to prevent nystagmus modifications. Bedside visual-oculomotor testing was also performed in all cases. Pathological signs were recorded using infrared video camera and stored on the hardware of a personal computer.

None of the patients underwent caloric stimulations; in our opinion these were unnecessary for the diagnosis because all patients complain of positional symptoms and they had paroxysmal vertical-torsional nystagmus.

All patients but one were addressed to carry out a neuroradiological investigation (cranial CT scan in 1 case and NMR in 4 cases) to rule out a central vestibular involvement. The remaining patient had a recent negative cerebral NMR scan executed, immediately before our indication, for recurrent positional vertigo.

At the first checkup, all patients were submitted to different types of physical manoeuvres for AC canalolithiasis: in 4 cases they had a “reversed” Epley's procedure [10]; in the remaining two we carried out a Vannucchi's manoeuvre proposed for AC canalolithiasis [14]. The latter procedure aims to impress a brisk deceleration to the particles, presumably located into the canal lumen near the ampulla, to move them out of it. The manoeuvre consists of the following steps: (a) patient is seated on the bed with the head rotated 45° towards the affected side; (b) the subject is rapidly laid down on the affected side; (c) after about one minute he is moved to the opposite side without changing the head position; (d) patient is brought to the seated position again. Patients were controlled during the same session (roughly after 30 minutes) and, at the most, after one week. 

During the second checkup we looked again only for spontaneous positional nystagmus, and when we found that nystagmus was qualitatively changed we adopted the appropriate physical therapies for that sign. 

Patients were evaluated once more at the distance, as maximum, of one week. 

## 3. Results

From the whole group of AC PPV we selected 6 patients because of a particular behaviour of their pathological signs during the period of observation and treatment. In fact, these patients, initially diagnosed and treated as having AC PPV (because they had a paroxysmal positional torsional down beating nystagmus in the head hanging positions) presented to the next control with a paroxysmal positional up beating vertical-torsional nystagmus, typical of a PC PPV of the opposite side. 

One patient had a cranial injury during her life. One patient was in treatment for hypertension. One patient had a Hodgkin's lymphoma but she got well at the time of our visit. The same patient had cervical injury and thyroid gland hypofunction. The remaining three patients did not have any considerable pathology in their histories.

Otomicroscopy was negative in all patients; audioimpedance testing was normal in three cases and consistent with presbyacusis in the other three subjects. 

All patients of our selection had essentially down beating nystagmus, when observed in one or more head hanging positions, using videooculoscopy or Frenzel lenses. Torsional component was not very evident in all cases and it was directed clockwise in all cases, but one. 

Nystagmus appeared in all cases with a very brief latency; it had a clear paroxysmal trend in 3 cases and a simil-paroxysmal behaviour in two. In one patient nystagmus appeared stationary being, otherwise, transitory. The mean duration of positional nystagmus was very long (one minute or more) and it was, in general, of small amplitude. 

In two subjects nystagmus was elicited in all three head hanging positions (Dix-Hallpike's positionings and central head hanging position); in the same two patients nystagmus was evident also in the side lateral positions. In one subject positional nystagmus was elicited only in one of the two Dix-Hallpike's positions, while in the other two nystagmus expressed into both Dix-Hallpike's positionings (not into the central head hanging). In the remaining patient nystagmus was observed into only one of the two Dix-Hallpike's positions and into central head hanging position. 

During the observation into the provoking position, nystagmus ended completely, although very slowly, in three cases: in the other three patients nystagmus lasted more than 2 minutes, although with a reduced amplitude. 

In only one case nystagmus reverted its direction coming up from the head hanging position. Repetition of the provoking position did not fatigue nystagmus in all cases but one.

On the base of such signs we initially diagnosed AC PPV of the left side in five cases and of the right side in the remaining one.

Patients had a “reversed” Epley's manoeuvre (4 subjects) or Vannucchi's manoeuvre (2 cases) to cure presumed AC PPV. 

In four patients, at the first checkup, during the same session, we surprisingly found positional nystagmus that had a reversed direction (both for the linear and the torsional components) with respect to the one seen during the previous observation. The same reversal of positional nystagmus was noticed at the second checkup for one patient and at the third visit for the remaining one (Table 1). 

In five patients clockwise down beating PPNy became counterclockwise up beating, as typical of a right PC PPV (instead of a left AC PPV); in the remaining subject counterclockwise down beating nystagmus reverted into a clockwise up beating nystagmus, as typical of a left PC PPV (instead of a right AC PPV).

Moreover, the actual signs showed the paradigmatic behaviour of a typical PC PPNy due to canalolithiasis. Thus, we submitted these patients to Semont's manoeuvre, during the same session, instead of performing physical therapy for AC of the opposite side. All the patients were symptoms- and signs-free at the next visits.

Three out of our 6 patients had already suffered in their recent past of a PC PPV: in all the three cases we documented a PC PPV of the same side we conclusively diagnosed. 

## 4. Discussion

In this study the prevalence of PC and LC canalolithiasis was the same as reported in literature. The estimated prevalence of AC PPV among our patients, as what diagnosed at the first visit, seemed higher with respect to what was commonly expected and reported by other authors [10, 15]. At that time, in fact, the one-year prevalence of AC canalolithiasis in our selection resulted of 3.2%, that is 15 cases out of 473 of PPV, each year, interesting one or more semicircular canals; such a prevalence increases up to 3.6% if we consider PPV strictly interesting only one semicircular canal (15 out of 412).

Initially, we included into the AC PPV group also the 6 patients that subsequently demonstrated a torsional up beating nystagmus, thus indicating a canalolithiasis of the posterior canal of the opposite side. The cases of these six patients have been, in our opinion, extraordinary, because nystagmus direction changed spontaneously and/or by the effect of physical therapy believed appropriated for AC PPV. This phenomenon lead us to try to look for a pathophysiological explanation of such a clinical behaviour. It is, obviously, quite impossible that six patients resolved their AC PPV of one side, developing a PC PPV of the contralateral side, immediately or within a few days. We therefore believed that PPV of these patients interested the PC of the contralateral side since the first visit, presenting, however, with a nonparadigmatic nystagmus pattern. 

Having an atypical nystagmus pattern, we had to rule out a central vestibular disturbance due to a brainstem and/or cerebellar dysfunction. Therefore, during the follow-up period, all the patients underwent a cerebral TC scan or NMR that was negative in all cases, especially with respect to expansive and vascular brainstem and or cerebellar lesions.

Moreover, patients' histories were consistent with a positional vertigo and negative for actual significant pathologies possibly related to vestibular signs and/or symptoms. Some of the patients had a previous diagnosis of PPV; clinical and instrumental audiological investigations were normal or age compatible in all cases. Feminine sex prevalence and medium age incidence were that of a PPV.

These data lead us to diagnose a peripheral paroxysmal positional vertigo. 

**Figure 2:**
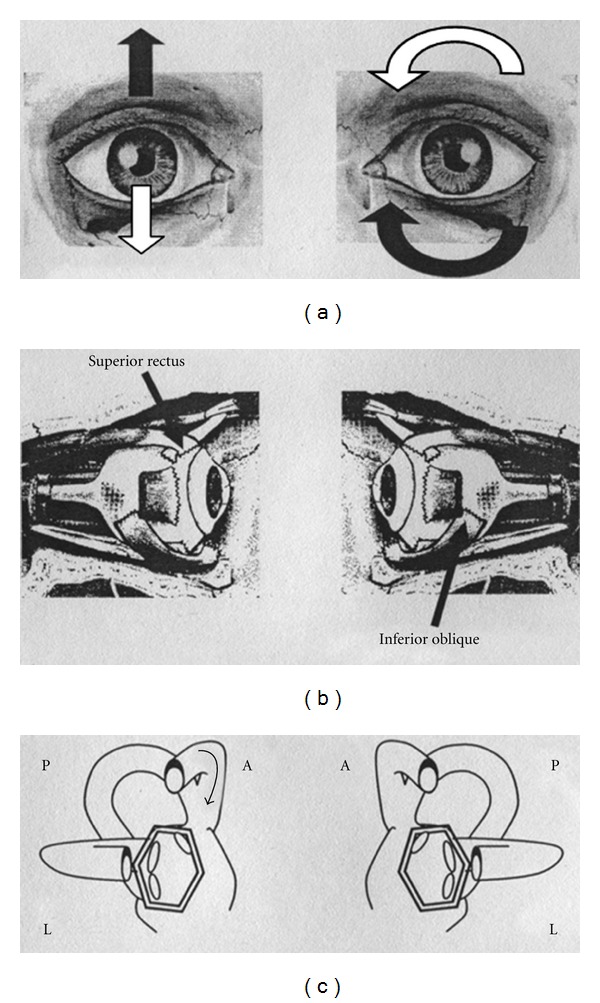
Paroxysmal positional nystagmus due to unilateral right anterior semicircular canal (AC) lithiasis (excitatory stimulus). (a): Black arrows indicate the direction of nystagmus slow phase in the two eyes; white arrows indicate the direction of nystagmus fast phase in the two eyes. (b): Arrows indicate the ocular muscles involved in nystagmus generation. (c): The two labyrinths; arrow indicate the endolymphatic flow within the interested canal. On the left side of the figure: the right eye and the right labyrinth; on the right side of the figure: the left eye and the left labyrinth. A: anterior semicircular canal; L: lateral semicircular canal; P: posterior semicircular canal.

**Figure 3:**
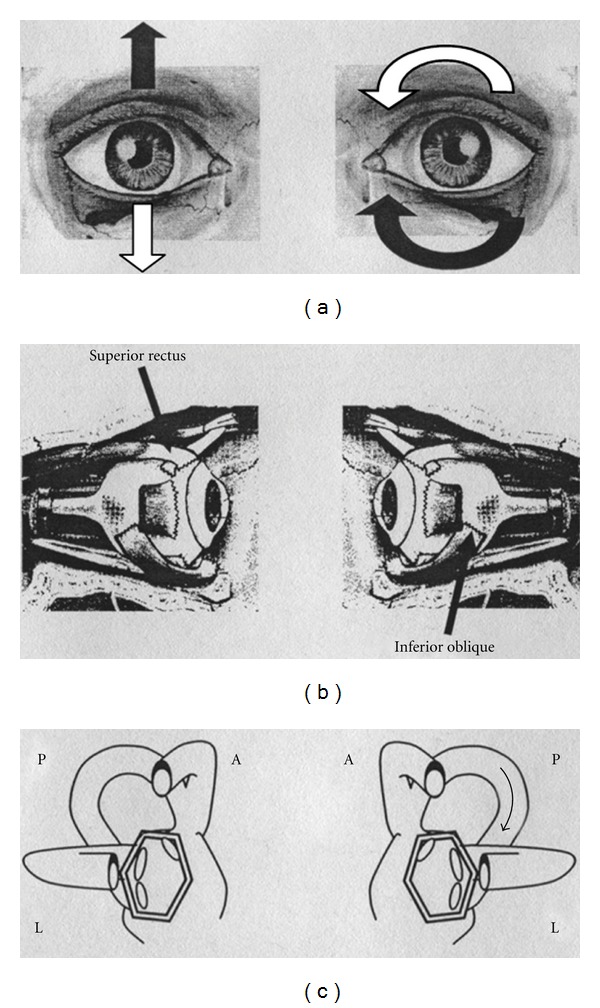
Paroxysmal positional nystagmus due to unilateral left posterior semicircular canal (PC) lithiasis (inhibitory stimulus). (a): Black arrows indicate the direction of nystagmus slow phase in the two eyes; white arrows indicate the direction of nystagmus fast phase in the two eyes. (b): Arrows indicate the ocular muscles involved in nystagmus generation. (c): The two labyrinths; arrow indicates the endolymphatic flow within the interested canal. On the left side of the figure: the right eye and the right labyrinth; on the right side of the figure: the left eye and the left labyrinth. A: anterior semicircular canal; L: lateral semicircular canal; P: posterior semicircular canal.

Morphological characteristics of positional nystagmus suggested, as a first instance, an AC PPV. In fact, paroxysmal or similar-paroxysmal nystagmus, elicited in Dix-Hallpike's and or head hanging positions, had a mixed torsional-vertical direction with the fast phase of the linear component directed downwards and the torsional element directed counterclockwise or clockwise, respectively, for right and left ACs of the considered side. Such a nystagmus could be in fact generated by the contraction of ipsilateral superior rectus and the contralateral inferior oblique muscles, considering an excitation of the anterior ampullary nerve. Nystagmus slow phase direction is directed upwards and clockwise or counterclockwise respectively for right and left ACs (Figure 2). Such a nystagmus could be, however, generated also by the inhibition of the posterior ampullary nerve of the opposite side, that drives the contraction of the same ocular muscles which in this case should be, respectively, contralateral and ipsilateral to the involved PC [16] (Figure 3). That is the reason why, unfortunately, it is impossible to distinguish the one to the other a priori: AC of one side and PC of the other one are, in fact, coplanar and work in a push-pull mechanism to obtain the same compensating ocular movement. 

Moreover, the two ocular movements cannot be distinguished looking upon nystagmus linear or torsional component potentiation into eccentric positions of gaze. In fact, the linear element is, that is, more evident in right position of gaze for right AC involvement and considering left PC stimulation. For the same reason, the torsional component is more evident in the right eye for the stimulation of the right PC and the left AC.

The two signs are undistinguishable also considering their intensities. Potential differences in nystagmus amplitude due to excitatory or inhibitory discharge of the ampullary nerves cannot be detected. In most cases, in fact, both PPNy due to AC lithiasis and positional nystagmus that we initially found in our group of patients do not reverse their direction when the patients came up from head hanging to the sitting position, rather persist with the same direction for a short time or immediately blow over. In the only one case in which we observed a reversal of positional nystagmus, when the patient was brought to the sitting position, we did not record any difference in nystagmus intensity elicited into the head hanging or into the sitting position. 

Since patients of our selection, believed to have AC PPV, developed a typical PPNy due to involvement of the PC of the opposite side only because of diagnostic movements or by practising a physical therapy, we had to understand how it could have happened. We first took in account a mechanical model from the moment that canalolithiasis is, most frequently, the pathophysiological mechanisms underlying PPV. Depending on where otoconial debris is initially localized, different nystagmic patterns are generated during head movements because of different endolymphatic currents produced by the clot movements into the semicircular canals.

The movement of some otoconial debris hypothetically localized into the region of PC adjacent to the common crus could produce, because of head movements, a nystagmic pattern that mimes that of AC canalolithiasis of the opposite side.

**Figure 4:**
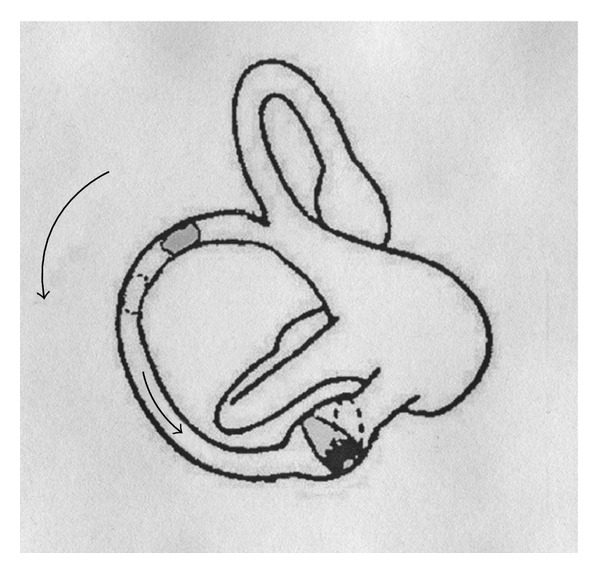
Left PC PPV due to nonampullary arm canal lithiasis. If the clot is localized into the non ampullary arm of the PC, when the patient is brought into the head hanging positions, the otoconial mass moves towards the ampulla; this movement produces a ampullopetal endolymphatic current and generates an inhibitory discharge of the posterior ampullary nerve. Thick arrow: movement of PC movement during positioning; thin arrow: direction of endolymphatic current after positioning; dashed lines: positions gained by the clot and the cupula, after positioning.

Assuming that the clot is localized into the nonampullary arm of the PC, near the common crus, when the patient is brought into the head hanging positions, the otoconial mass should move towards the ampulla; this movement would produce an ampullopetal endolymphatic current thus generating an inhibitory discharge of the posterior ampullary nerve. Such a stimulus generates an ocular movement identical to the one described before: a paroxysmal or similar-paroxysmal down beating vertical torsional nystagmus. The involved PC will be recognized by the direction of the fast phase of nystagmus torsional component (clockwise or counterclockwise, resp., for right and left PC) more evident into the eye ipsilateral to the interested PC (Figure 4).

The impressive change of nystagmus direction obtained by means of diagnostic movements and/or physical treatment could simply result from a displacement of the debris into the canal lumen. In fact, diagnostic or therapeutic movements could provoke the displacement of the clot from the nonampullary arm towards the ampullary end of the PC, reproducing the anathomopathological condition of a typical PC canalolithiasis. With such a initial localization, head hanging positions generate the well-known torsional-up beating nystagmus (counterclockwise or clockwise, resp., for right and left PCs). 

**Figure 5:**
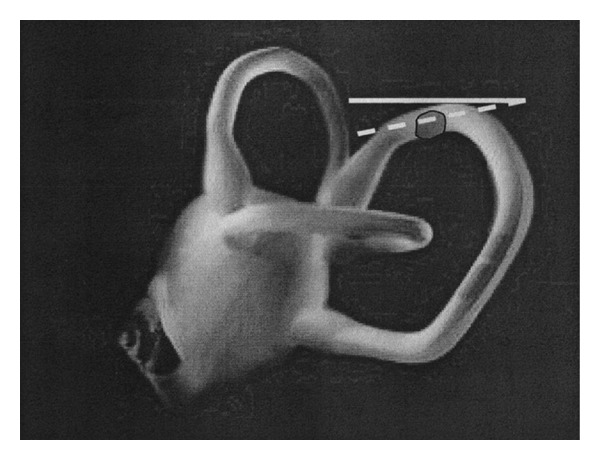
Left PC PPV due to nonampullary arm canalolithiasis. Nystagmus does not reverse its direction when the patient returns into the sitting position: the lack of reversal could be due to the reduced movement of the clot in a restricted tract of the PC that, is in a roughly horizontal plane. Solid line: horizontal plane; dashed line: plane corresponding to that of nonampullary arm of PC in the sitting position.

Positional torsional down beating nystagmus observed in our patients did not reverse its direction when the patient returned into the sitting position, after the head hanging one; sometimes nystagmus disappeared and sometimes it continued for a while, then vanished. The lack of reversal could be due to reduced movement of the clot in a restricted tract of the PC that, besides, moves in a roughly horizontal plane, when the patient is brought back to the sitting position (Figure 5). 

Recently, another pathogenetic hypothesis has been introduced to explain some atypical patterns of horizontal direction changing nystagmus found in patients with LC PPV [9, 17]. The latter theorizes that, because of some metabolic factors, the cupula of semicircular canals would become heavier or lighter with respect to the endolymph. Such a difference in cupular weight should make the canal receptors sensitive to gravity, with a modality different from that of a canalolithiasis. Horizontal direction changing nystagmus due to heavy or light cupula reverses its direction varying the horizontal position of the head and shows a “null point” when the head reaches a position so that gravity vector does not influence the two cupulae.

In our group of patients we noticed only one cases of a clear direction changing nystagmus, positional nystagmus being, surprisingly, monodirectional at most, showing not reversal when patients were brought up from the Dix- Hallpike's to the sitting position. Moreover, moving the patient's head onto the vertical plane, performing Dix-Hallpike's positioning test, we never found a position in which nystagmus was no more detectable; torsional down-beating nystagmus rather continued a little even if the patient remained into the sitting position. In our opinion, light (in our cases) cupula theory is not convincing also because of the unexpected modification of nystagmus direction observed, sometimes, during the course of a single observation. Similarly to what happens for postalcoholic nystagmus, a modification of cupular density should take place some hours before to change or dash, and nystagmus should not change so dramatically in a few minutes. 

## 5. Conclusions

Since its first detailed description in the earlier fifties until the present, PPV has been more and more studied and understood in all its typical and atypical aspects. Besides typical patterns canalolithiasis theory, the pathogenetic mechanism underlying most of PPV, has been often explanatory for some atypical nystagmus found in PPV that could have been attributed before to central vestibular disturbances. Moreover, atypical positional nystagmus can sometimes be transformed into paradigmatic benign paroxysmal positional nystagmus, simply by means of diagnostic or therapeutic manoeuvres. The canal clot in fact could move into the semicircular canals by the effect of the changing gravity vector. 

That is what, sometimes, happens in LC PPV initially presenting with an apogeotropic bidirectional nystagmus: only by means of therapeutic or diagnostic movements, nystagmus can reverse its direction becoming geotropic in both lateral side positions. 

In our selected cases we hypothesized a similar mechanism to explain why our patients, initially presenting with a nystagmic pattern consistent with AC PPV, came back to the next checkup with a typical PC PPNy of the opposite side. The initial apogeotropic vertical-torsional nystagmus changed, by means of physical therapeutic procedures or simply repeating positions, into typical geotropic PPNy.

As for LC PPV we can theorize that also PC PPV could manifests with two different nystagmic patterns; one of them, more frequent, is that with a geotropic vertical-torsional PPNy; the other one, more rare, is that with a vertical-torsional nystagmus that in the provoking positions is directed downwards, for that, away from gravity. In analogy with LC PPV, the latter form could represent the apogeotropic variant of PC PPV.

From 2006 to present, studying about 5000 vertiginous patients every year, we collected roughly 2400 cases of BPPV, of which 150 showed a nystagmic pattern similar to that described in the reported pilot group with an incidence of 6.25%. Therapy for such a PC PPV variant should be different from that of an AC PPV or PC PPV presenting with geotropic nystagmus. To cure PC PPV apogeotropic variant we are testing specific manoeuvres that aim to resolve symptoms with a single treatment approach or, at least, to convert nystagmus into the typical one. 

## Figures and Tables

**Table 1a:** (a)

	First checkup				
Patient	Provoking manoeuvre(s)	PPNy	Reversal of PPNy	SC hypothesized	Therapy
1	Right DH-left DH-HHP- right SLP-left SLP	CW-DB	Absent	Left AC	“Reversed” Epley's manoeuvre
2	Right DH-left DH-HHP- right SLP-left SLP	CW-DB	Absent	Left AC	“Reversed” Epley's manoeuvre
3	Left DH	CW-DB	Absent	Left AC	“Reversed” Epley's manoeuvre
4	Right DH-left DH	CW-DB	Absent	Left AC	Vannucchi's manoeuvre
5	Right DH-left DH	CW-DB	Absent	Left AC	Vannucchi's manoeuvre
6	Right DH-HHP	CCW-DB	CW-UB	Right AC	“Reversed” Epley's manoeuvre

**Table 1b:** (b)

	After 30 minutes			Second check-up		
Patient	PPNy	SC hypothesized	Therapy	PPNy	SC hypothesized	Therapy
1	CCW-UB	Right PC	Semont's manoeuvre	Absent	—	—
2	CCW-UB	Right PC	Semont's manoeuvre	Absent	—	—
3	CW-DB	Left AC	“Reversed” Epley's manoeuvre	CW-DB	Left AC	“Reversed” Epley's manoeuvre
4	CCW-UB	Right PC	Semont's manoeuvre	Absent	—	—
5	CW-DB	Left AC	Vannucchi's manoeuvre	CCW-UB	Right PC	Semont's manoeuvre
6	CW-UB	Left PC	Semont's manoeuvre	Absent	—	—

**Table 1c:** (c)

	Third check-up			Fourth check-up		
Patient	PPNy	SC hypothesized	Therapy	PPNy	SC hypothesized	Therapy
1	—	—	—	—	—	—
2	—	—	—	—	—	—
3	CCW-UB	Right PC	Semont's manoeuvre	Absent	—	—
4	—	—	—	—	—	—
5	Absent	—	—	—	—	—
6	—	—	—	—	—	—
